# Comment on Chow, J.W.; Stokic, D.S. Longitudinal Changes in Temporospatial Gait Characteristics during the First Year Post-Stroke. *Brain Sci.* 2021, *11*, 1648

**DOI:** 10.3390/brainsci12080996

**Published:** 2022-07-28

**Authors:** Janis J. Daly

**Affiliations:** 1 Brain Rehabilitation Research Center (BRRC), NFSG Malcom Randall VA Medical Center, Gainesville, FL 32608, USA; janisaly@ufl.edu or jjd17@case.edu; 2 College of Public Health and Health Professions, University of Florida, Gainesville, FL 32608, USA; 3 Department of Neurology, Case Western Reserve University, Cleveland, OH 44016, USA

## 1. The Limitation of the Gait Speed Measure in Neurorehabilitation

The field of neurorehabilitation has moved considerably beyond a narrow use of gait speed. Studies and reviews are available of sophisticated and direct measures to identify mechanisms underlying an increase in gait speed (e.g., [1]), as well as descriptions of the clinically deployable methods for doing so (e.g., [2]). In fact, others have stated that ‘[spatial or] temporal measures’ including step length, stride length, and cadence “are **not representative** of factors that are independent of the changes in walking speed, and should be considered ‘intermediary’, but they are not true mechanistic measures. Due to a dependent relationship, [these spatial or] temporal measures may not give insight into the true mechanism of change”. Further, in an example from their extensive review, they concluded that “current data do not conclusively demonstrate that asymmetry measures reflect recovery, and one must continue to apply restraint in considering asymmetry measures to be the mechanism of change” [1].

Contemporary clinical and clinician-science professionals are employing a sophisticated array of actual gait coordination measures (for example, kinematics and kinetics), as well as assessment of balance and functional activities in administering patient care and in designing research studies. Measures can be efficiently administered by expert clinicians, reducing the patient burden to a minimum. More mechanistically based information is what is required in order to advance the field of gait rehabilitation in both clinical practice and in research. Exciting, new, easily deployable technologies for these measures are predicted for the near future [2]. As a result, caution is advised because the minimal group of measures presented in the current paper would not be adequate in serving as the basis of an efficacious treatment plan nor in understanding treatment response [1,3].

### 1.1. Underlying Factors of Normal, Chosen-Speed Coordinated Gait Pattern

The normally coordinated, chosen-speed gait pattern in human adults is based on numerous factors including the following: muscle activations and deactivations, temporally coordinated movements of limb joints, precisely and relatively-timed coordination of multiple limb joints, and balance control [4], as well as all of those factors together, precisely timed to exploit biomechanical energies (potential (PE) and kinetic energies (KE) of the center of mass and limb segments (e.g., thigh and shank) [5].

### 1.2. Considerations on the Background and Meaning of Gait Speed

The measure of gait speed has a long history in the field of neurorehabilitation, dating back to a bygone era when there were no sophisticated motion capture systems to acquire gait kinematics and gait kinetics. Because it is a cheap and easy measure to acquire (e.g., a tape measure and a watch), the field used gait speed as a measure of recovery for stroke survivors. However, reduced gait speed after stroke is a compensatory strategy in response to impairment in the underlying mechanisms of gait coordination and balance. Therefore, gait speed does not provide any information as to the dyscoordination or imbalance inherent in the impaired gait pattern after stroke. Moreover, in a stroke survivor, an increase in gait speed over time can simply be the result of ‘speeding up’ a dyscoordinated gait pattern, rendering the individual even more unsafe than when using the slower gait pattern; for those with sufficient clinical and research experience, this is a situation that has been encountered. Therefore, when making decisions regarding the need for and/or the efficacy of treatment, it is critical to acquire, analyze, and consider gait dyscoordination facts (defined in Section 1.3) and impaired balance facts in order to provide the proper context within which to assign meaning to a measure of gait speed. 

### 1.3. A Gait Coordination Measure by Definition must Incorporate Simultaneously Both a Temporal and Spatial Component within a Given Measure

In contrast, the current paper provides an array of unidimensional temporal measures (e.g., gait speed, cadence, time period in various stance sub-phases, and symmetry indices). They provide three unidimensional spatial measures. They do not provide an actual gait coordination measure, which, by definition [6] requires the simultaneous components of both a spatial component and a temporal component within the given measure. In fact, normal gait coordination is considered a ‘higher-order property’ of the locomotor system [6], p. 213, having simultaneously both ‘spatial and temporal components’ [6]. These precise combined spatial/temporal components are sequentially executed across the gait cycle [6] resulting in a gait pattern of optimal energy cost, both biomechanically [5] and physiologically [4]. A unidimensional temporal or unidimensional spatial measure is not considered a gait coordination measure, because it is simply missing a crucial component of coordinated movement. To emphasize the salient point: for a measure to be considered a measure of gait ‘coordination’, it must contain both a ‘temporal component and a spatial component’ [6]. An example of a measure of gait coordination is ‘knee flexion angle at toe-off’; in this case, knee flexion angle is the spatial measure and toe-off is the temporal (time of) event during the gait cycle. The field of neurorehabilitation has become more sophisticated in considering the gait coordination and balance deficits underlying stroke gait deficits. Neither unidimensional temporal nor unidimensional spatial measures provide the necessary information needed to support current clinical practice or advance the field.

### 1.4. Example of Failure to Extract Cogent Meaning of Gait Speed Change in the Absence of Gait Coordination or Balance Measures

In the current paper, there is no gait coordination information that would be required to understand the meaning underlying any gait speed change reported in the subjects. The danger in this situation is observable in the results and conclusions given in Section 1.4.4, arising from conducting the basic and logical descriptive analysis in the three steps below, in which their five subjects are evaluated (the only five subjects who exhibited a measurable increase in gait speed from 6 to 12 months post-stroke).

#### 1.4.1. Step One; Eliminate from Further Analysis, any Subject with Only Measurement Error (below the MDC Threshold (i.e., 19/24 subjects (79%))

In simple descriptive analysis, we can apply the most recent and precise minimum detectible change (MDC) thresholds [7] for gait speed. In general, the MDC is a threshold for any measure, below which is simply measurement error. The most recent, precise MDC thresholds for stroke gait speed are provided by Levitt & Sykes [7]. Applying the MDC thresholds to the original Table 1 in the associated Chow et al. paper, we can accurately observe that 19/24 subjects (79%) had no increase in gait speed from 6 to 12 months. That is, their difference score from 6 to 12 months did not reach beyond only measurement error (i.e., within the threshold for measurement error for gait speed). The fact is, then, that there were only five subjects (5/24; 21%)) who had a measurable increase in gait speed from T1 to T2, that is, from 6 to 12 months post-stroke (subjects: S2, S8, S24, S35, S38). 

#### 1.4.2. Step Two; Extract Limb Coordination Difference Values (FM-LE) (Difference from 6 to 12 Months) for the 5/24 Subjects Who Had a Measurable Change in Gait Speed

According to the data from Table 1 of the original paper, there were five subjects who did have a measurable increase in gait speed. From the original Table 1, we can extract, their respective performances on the Fugl-Meyer limb joint coordination measure (FM-LE) at both 6 and 12 months, and calculate their respective individual change in FM score.

#### 1.4.3. Step Three; Apply the Known MDC for the Limb Coordination Scale (FM-LE Measure) to the Five Subjects with a Measurable Increase in Gait Speed

In the basic descriptive analysis for each of these five subjects, we can extract the FM-LE value at 6 months and 12 months and find the difference value. Using that difference value in FM-LE, from 6 to 12 months, we can apply the most recent information on minimum detectible change (MDC) thresholds for the Fugl–Meyer limb joint coordination measure (FM-LE). According to Hiengkaew et al. [8], the minimum detectible change (MDC) for the FM-LE measure is 4.0 points. In that light, inspecting the difference value for each of these five subjects, we can note that none of them had a quantifiably measurable increase in FM-LE. The specific information is as follows for the FM-LE difference score from 6 to 12 months for the five subjects:

For two subjects, the FM-LE difference score worsened (S35, −7 points; S38, −6 points);

For one subject, no change (S24, 0 points);

For two subjects, the FM-LE difference score was 3.0 or 2.0 points but did not reach the MDC of 4.0 points (S2, S8).

#### 1.4.4. Step Four, Conclusions Drawn from the above Analyses: Inability to Accurately Interpret Gait Speed Change due to a Dataset That Is Limited to Unidimensional Measures

Limb coordination (FM-LE) did not improve for the above five subjects. This information begs the question: how did these above five subjects show an increase in gait speed? We can speculate that the subjects ‘speeded up’ their dyscoordinated pattern of movement. If so, this would argue for urgent intervention and gait training to recover a more coordinated gait before a catastrophic fall occurs. Of course, we do not know the meaning of the gait speed increase because, in the absence of true gait coordination or balance measures, we do not know through what means the increase in gait speed was accomplished in these five individuals. 

### 1.5. Call for Sophisticated Study of Gait Coordination Changes in Response to Promising Gait Training and Interventions

This is a call to clinicians and research scientists to continue, according to the most recent advances in the field, to acquire measures of balance and actual gait coordination measures, with each gait coordination measure composed of both a temporal and a spatial component. Let us take care not to be complacent in our terminology; rather, let us be vigilant so that we can discriminate between the following:

**A. Misleading terminology**: Use of the term ‘temporo-spatial measures’, meaning simply an array of unidimensional measures of either temporal or spatial characteristics.

**B. Accurate terminology**: Use of the term ‘temporospatial measure’, meaning an actual measure of gait coordination composed of both a temporal and a spatial component within the same given measure.

This is a call to acquire actual gait coordination measures by whatever means is practically available. For those who may not be familiar with current clinical practice, examples of balance measures include the Berg Balance Measure [9,10] and the Functional Gait Assessment [11,12,13], each requiring only a short time for administration; and in clinical practice, these are not considered burdensome to patients. Observational gait coordination measures are available for a number of neurological diagnostic categories, and psychometrics have been well tested for at least one gait coordination measure (e.g., for stroke [14,15,16,17,18], and for multiple sclerosis (MS) [19,20,21]). The gold standard of quantitative gait kinematics and kinetics is data acquisition through motion capture and force plate systems, and there are numerous commercially available companies that can provide and install these systems. In our experience, only 15 min of a patient’s time is required in order to apply the markers used in the most basic motion capture systems. Analysis of these data is not difficult with the software available in these contemporary times [2]. Acquisition and analyses of kinematic and kinematics data during testing of new gait training interventions is a sophisticated path forward in neurorehabilitation research. 

## 2. Invalid Methods in ‘Individual Analysis’ from 6 to 12 Months Leads to False Statement of Result

### 2.1. False Statement and Corrected True Statement

The authors have made a false statement in the current paper as follows: “10/24 (42%) had a statistically significant increase in gait speed from 6 to 12 months post-stroke.” The corrected true statement is as follows: 5/24 (21%) had a measurable increase in gait speed from 6 to 12 months post-stroke.

The logic and evidence supporting the corrected statement are provided in Section 2.2 and Section 2.3 below.

### 2.2. Order of Analysis in Good Lab Practice (GLP)

Prior to conducting statistical analysis on a dataset, it is a basic standard of good lab practice (GLP) to inspect the dataset for integrity and to conduct any relevant descriptive analyses. From the above simple descriptive analysis (Section 1.4), we have observed that only 5/24 (21%) individuals exhibited a quantifiable (measurable) increase in gait speed from 6 to 12 months. For the remainder, (19/24; 79%), there was either no change or a decrease in gait speed from 6 to 12 months. That is, 19/24 subjects had either a decrease in value from 6 to 12 months or their difference value fell within only measurement error for their respective gait speeds (‘no change group’; 19/24 (79%)) [7].

However, without considering that 19/24 subjects had no measurable increase in gait speed, the authors loaded the data from each of these 19/24 no-change subjects into a t-test to ‘see if there was a statistically significant change’. This is not a logical GLP process. The proof that it is not a logical nor a GLP practice is that there is not an accurate manner in which to describe the result. That is, one would be required to state that ‘those with no quantitatively measurable change had a statistically significant change’. That statement is not logical.

The take-away point is as follows: given the illogic and invalidity of the procedure and its result, it is important that the multiple statements to that effect are disregarded (that is, disregard the following: ‘10/24 (41%) had a statistically significant increase in gait speed’ from 6 to 12 months’.

### 2.3. Required Basic General t-Test Model Assumptions Are Breached in the Presence of Correlated Gait Cycles within Individual-Subject Data Points at 6 Months and in the Presence of Correlated Gait Cycles within the Data Points at 12 Months

Correlated gait cycle data are unsuitable for *t*-test models. As we are all aware, there are specific methods for conducting individual subject data analysis. One cautionary note is that repeated measures of data from a single subject can contain correlated data (serial dependency [22]), which can interfere with and invalidate statistical *t*-test models that cannot account for or correct for such lack of data integrity. Serially dependent data can give biased or false results when loaded into a *t*-test model.

In the current paper [23], the authors state that subjects walked 4–5 times across a walkway (‘4–5 passes’), from which they collected 10–25 gait cycles from a given subject. This means that for any given subject, sequential gait cycles were collected, ranging from 2 to 6 sequential gait cycles, depending upon the number of passes and number of cycles extracted. As is obvious, the gait speed of a particular gait cycle is influenced by its preceding cycle; and similarly, the given gait cycle influences its subsequent gait cycle. This situation of serially dependent data within the 6-month or within the 12-month data sets results in probable interference of the proper functioning of a t-test model, invalidating the results for potential bias and patently false results.

The take-away point is as follows: the use of t-test analysis of gait cycles of any given individual subject comparing his/her 6-month serial gait cycles with his/her 12-month serial gait cycles is highly suspect and may be patently false.

## 3. Conclusions

Considering the above two main points in Section 2, the statements in this paper of 10/24 (42%) having a ‘significant’ increase in gait speed should be considered suspect, at best, and potentially false. To summarize, the potentially false statement is as follows: “10/24 (42%) had a statistically significant increase in gait speed from 6 to 12 months post-stroke. The corrected true statement is as follows: 5/24 (21%) had a measurable increase in gait speed from 6 to 12 months post-stroke.
